# Relationship between Eye Blink Frequency and Incremental Exercise among Young Healthy Men

**DOI:** 10.3390/ijerph19074362

**Published:** 2022-04-05

**Authors:** Wojciech Paśko, Emilian Zadarko, Tomasz Krzeszowski, Krzysztof Przednowek

**Affiliations:** 1 Institute of Physical Culture Sciences, Medical College of Rzeszow University, Rzeszow University, 35-959 Rzeszow, Poland; wopasko@ur.edu.pl (W.P.); ezadarko@ur.edu.pl (E.Z.); 2 Faculty of Electrical and Computer Engineering, Rzeszow University of Technology, Al. Powstancow Warszawy 12, 35-959 Rzeszow, Poland; tkrzeszo@prz.edu.pl

**Keywords:** eye blink, time of closed eye, blinking eye detection, incremental exercise, fatigue

## Abstract

The aim of the study was to verify the correlation between the frequency of blinking and aerobic physical exercise. The research subjects were 13 healthy man aged 23.3 ± 1 year. Measurements of the blink rate and eye closure times were performed during a progressive aerobic test on a cycle ergometer. During the test, power was gradually increased every minute by 25 W, starting from 50 W. Data acquisition involved using a GoPro camera mounted to the helmet of the research subject. The test continued until the research subject refused to continue. The subjects did not know the goal of the test, in order to ensure objectivity and obtain natural results. The largest number of statistically significant differences was observed between the initial stages and 250 W, as well as between 250 W and 325 W. The analysis showed no significant differences in blink rate, eye closure time, and single blink time in terms of heart rate ranges. Regression models were also determined for eye closure time, blink frequency, and single blink time. The analysis showed that blink frequency and eye closure time were determined by a group of factors (the value of cycle ergometer load power, heart rate, body weight, adipose tissue mass, fat-free mass, and total body water and body surface ratio).

## 1. Introduction

Blinking can be classified as voluntary, i.e., intentional and controlled by a person, or involuntary, i.e., performed unconsciously and spontaneously. The duration of an involuntary blink is approximately 0.25–0.75 s [1]. Blink rate is determined by numerous factors, and on average it is 2 to 50 blinks per minute [2]. People blink less frequently while performing visual tasks such as reading than while resting quietly, whereas during other activities such as talking, etc., the blink rate increases and can be observed between phrases and at the ends of sentences [3]. The blink rate decreases during cognitive processes, i.e., processing of information by the mind, which leads to the conclusion that blinking may interfere with cognitive processes [4]. Moraleda [5] claims that eye tracking allows for recognition of the cognitive functions that a potential user can perform. Maffei and Angrilli [6] observed that the greater the concentration on a performed task, the lower the blink frequency. Increased blink rate may also be caused by eye dryness, which may be due to a health condition or specific climatic conditions [7]. Certain diseases, such as Parkinson’s disease, reduce the spontaneous blink rate [8]. The blink analysis was also used to detect concussion in soccer players who played the ball using their heads [9]. The study carried out by Wylęgała [10] showed the positive effect of physical activity on the physiology of the eye through increased retinal blood flow, and it is considered that physical activity prevents common eye diseases.

Fatigue can be fundamentally classified as mental (central) and physical (peripheral). Mental fatigue results in reduced concentration and disturbs the retrieval of information stored in memory, while physical fatigue reduces the capacity to perform physical work due to decreased muscle power [11]. Fatigue is often measured using the Borg scale, which offers basic information about the degree of intensity of physical exercise [12]. The Borg scale is a subjective method and cannot be used during exercise. The following parameters can be used to monitor the intensity of exercise and to assess the degree of fatigue: heart rate, lactate threshold, and maximum oxygen consumption, all of which require specialist equipment [13]. The observed effort and intensity of exercise can be also assessed through electromyographic (EMG) analysis of the face because heart rate (HR) correlates with the activity of facial muscles during high-intensity exercise [14,15].

A number of works have been carried out on the analysis of the impact of fatigue on the frequency of blinking [16,17,18,19,20,21,22,23]. This research could lead to the development of innovative non-contact and non-invasive methods of measuring effort and fatigue. For example, Haq and Hasan [16] showed that the level of attentiveness correlates with blink rate, and therefore variations in the frequency and duration of blinks can be used in the diagnostics of fatigue or drowsiness. In another paper [22], in order to detect tiredness in an employee working in front of a computer, the measurement of blink frequency was used. The analysis of blink frequency was also performed to identify drowsiness in drivers [18,19]. Islam et al. [19] utilized facial landmark detection for eye detecting and eye aspect ratio to detect blink. They observed the maximum frequency of blinks in the morning, and a gradual decrease in blink frequency throughout the day, with the lowest blink frequency recorded at midnight. In turn, in [17], Zhang et al. presented an algorithm that measured both heart rate and blink frequency using a contactless technique based on a smartphone. During the study carried out by Wylęgała et al. [20], the blink frequencies measured during rest and exercise had different intensities. During moderate-intensity exercises, blink frequency was lower than during rest, while the measurements performed during high-intensity exercises showed that the blink frequency was higher. The increase in blink frequency during exercise was also observed in the study carried out by Chiviacowsky et al. [21].

Another method for evaluating effort and assessing fatigue in a contact-free and non-invasive manner can be an observation of facial expression [24,25,26,27,28,29,30]. For example, Khanal et al. [27] proposed that a method of determining the intensity of exercise consists of establishing orientation points on the face and monitoring the changes in distance between them during exercise and progressive fatigue. The observed distances can help classify the intensity of exercise and determine the level of fatigue. Irani et al. [25] proved that observation of facial movements allows one to measure the heart rate using computer techniques since blood circulation in the head is associated with temporary facial movements. In the paper [28], authors showed that facial analysis during physical exercise indicates that the blink rate increases with time and with heavier load, while mouth opening increases to facilitate oxygen uptake.

To gain more knowledge on observations related to the level of tiredness during physical exercises using contactless methods, the authors carried out an analysis of the relationship between tiredness and blink frequency. The purpose of the paper was to analyse how physical tiredness affects blink frequency and eye closure time. The experiment presented here was conducted to see if blink parameters change when fatigue occurs. This study aims to demonstrate that fatigue levels can be determined by observing an athlete’s blinks. This method is non-invasive, and the assessment appears to be objective and feasible during athletic exercise. If this relationship is verified, it may help to observe, among other things, an athlete during training or competition. The research is basic research and has not been directly applied at this stage yet. The study involved a standardised stress test with heart rate measurement and precision recording of the upper part of the face using a high-speed camera. Blink frequency and eye closure time were also compared with body composition. The comparison was designed to test whether blink frequency and eye closure time could be affected by body composition and weight. The measurements were carried out during a progressive aerobic test.

## 2. Materials

The study involved thirteen males, aged 23.3 ± 1 years. Each subject started the test in a rested condition and on a voluntary basis. Before the progressive test, the subjects’ body weight was measured, and their body composition was analysed using the bioelectricalimpedance method (Table 1). The obtained HR${}_{max}$, HR${}_{rest}$, Power${}_{\bar{x}}$, and Power${}_{max}$ values are also included in Table 1 to characteristics the study group. HR${}_{rest}$ was measured before the test when the subject waited 1 min on the cycle ergometer and was ready to perform the test. HR${}_{max}$ means the maximum heart rate reached during the test. Power${}_{\bar{x}}$ means the average power per kilogram of body weight during the entire test. Power${}_{max}$ indicates the maximum power that was per kilogram of body weight during the entire test.

Only adult males who were rested and who did not have a health problem took part in the study. Written informed consent was obtained from the participants prior to the study, and an interview was conducted to exclude any individuals who might interfere with the results, such as individuals with eye diseases or nervous tics. None of the participants were treated pharmacologically, and none were injured during the study. The scope and project research were assessed by the Ethics Committee of the University of Rzeszow/Poland (resolution 22 April 2021).

## 3. Methods

**Figure 1:**
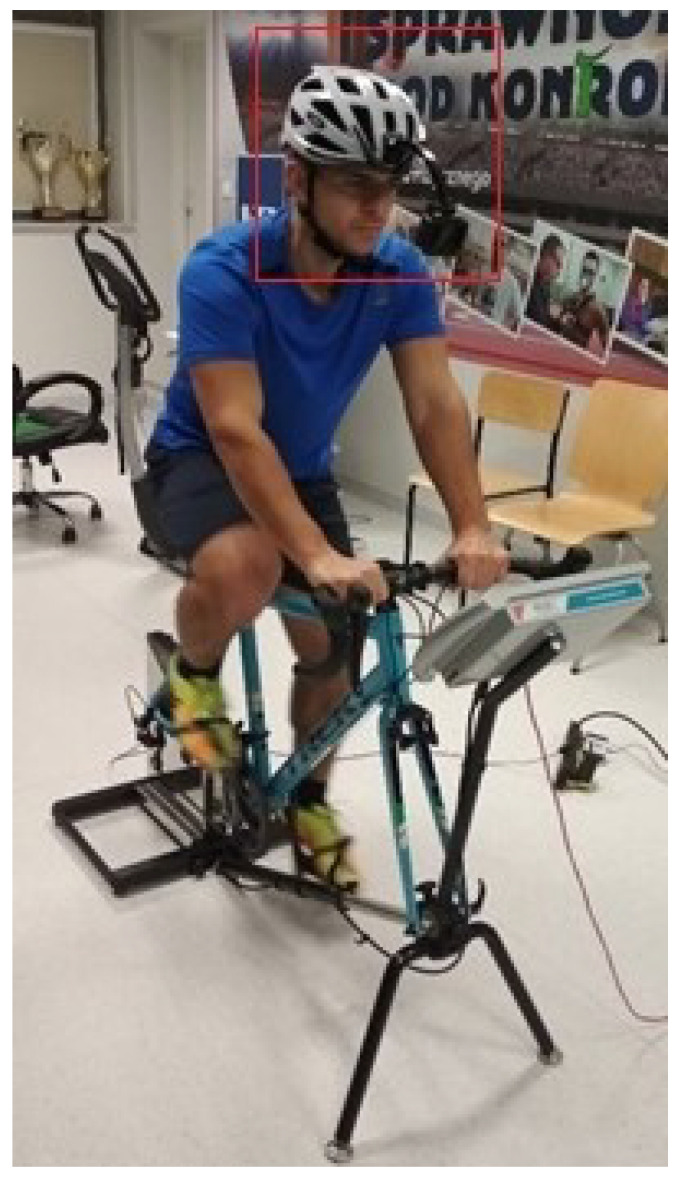
Stand for carrying out the oxygen progressive test.

The test was conducted under laboratory conditions, where the temperature was 19 ${}^{\circ }$C, and the lighting was constant during all measurements. During the test, windows and doors were closed to avoid wind gusts and to eliminate other external factors affecting blink frequency. There was only one subject and two investigators in the room so that the subject was not distracted by the others. The progressive test of oxygen use was conducted using a bicycle ergometer with Cyclus2 software, with the latter being used to establish the appropriate test protocol and the analysis of data. To capture the blink rate, a GoPro camera was attached to the helmet of the subject and directed towards their eyes (Figure 1), set to register the images during the test at a resolution of 760p and a with frame rate of 60 fps.

**Figure 2:**
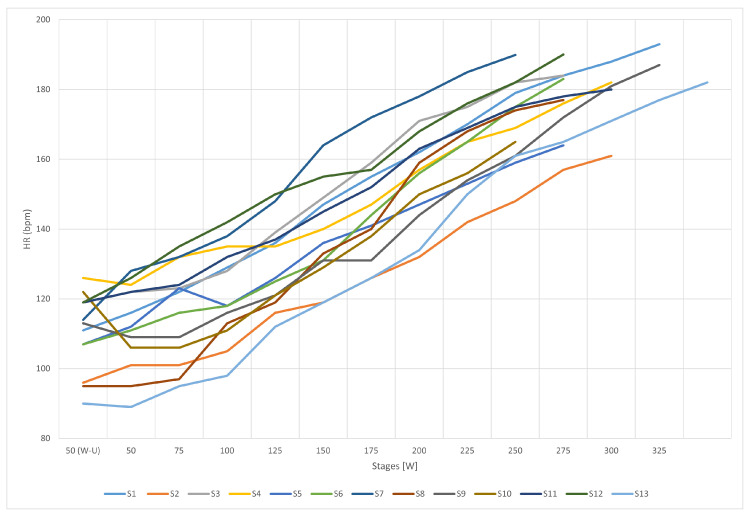
The relationship between heart rate and stages; S1:S13—research subjects.

The heart rate was measured during the test by a POLAR heart rate sensor in combination with a Cyclus2, and the results were registered in 1-min increments. The first stage corresponded to a warm-up and comprised exercising for 2 min with a workload of 50 W. After the warm-up, the power was increased in increments of 25 W per minute. The exercise test was conducted until the subject could not continue the workout at the current power level. It is worth noting the intensity of exercise (Power [W]) corresponds to the level of fatigue (Figure 2). The subjects did not know the goal of the test, in order to ensure objectivity and obtain natural results [31].

**Figure 3:**
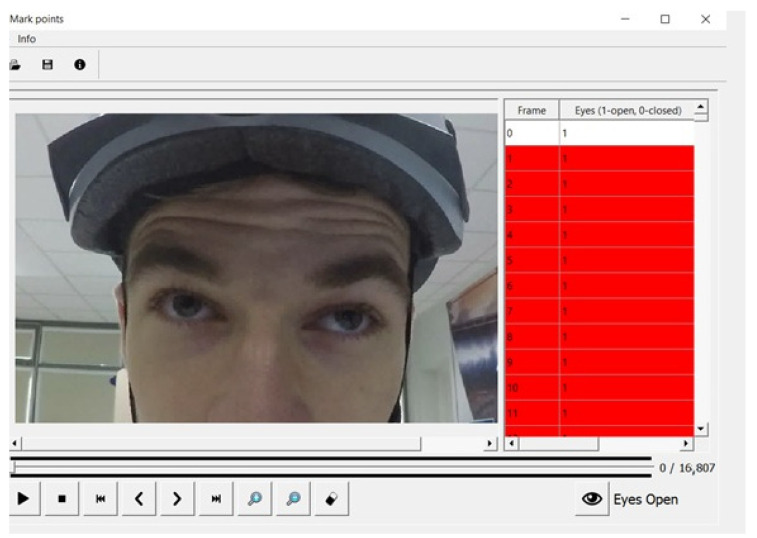
Eye-blink analysis in MarkBlinks programme.

**Figure 4:**
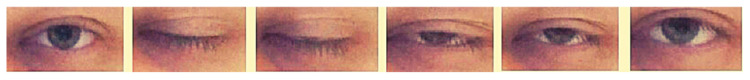
Eye blink.

The records were transferred to a computer, where the frame rate was reduced to 20 fps. The time of the closed eye was determined by summing the number of frames in which the closed eye occurred. One frame corresponded to 0.017 s. The MarkBlinks program (Figure 3) was prepared by the authors for the purpose of counting blinks, frame by frame. A 1 signified “eye open”, and a 0 signified “eye closed”. Pictures 1 and 6 show open eye, and pictures 2–5 show closed eye (Figure 4). The “eye open” condition was set when no more than half of the pupil was obscured; otherwise, the eye was classified as closed. This was a subjective assessment by the person who checked the recordings.

### Statistical Method

The results of blinking at individual stages and a given level of intensity were presented using the descriptive characteristics (number, mean, standard deviation, and median). Normal distribution was verified using Shapiro–Wilk test. Due to the fact that most of the analysed variables do not have normal distribution, non-parametric tests were used. The Wilcoxon test was used to show statistically significant differences between the selected heart rate ranges and blinking. The Friedman test was used to compare means in several dependent groups (individual power-load values). This test was used to detect differences in treatments across multiple test attempt. The Friedman test is the non-parametric equivalent of analysis of variance in a repeated-measures design.

Box-plots were used for the graphical analysis and the statistical significance of the differences was determined. The purpose of using progressive multiple regression was to assess the influence of individual variables on blinking parameters during progressive exercise. All calculations and analyses were performed using the GNU R program.

## 4. Results

Table 2 presents the blink rate and eye closure time at individual stages of the progressive test. The arithmetic mean of the blink rates at different stages ranged from 19.8 to 27.3 blinks. The highest average blink frequency (27.3 ± 20.0 blinks/min) was observed at the 250 W stage, while the lowest average blink frequency (19.8 ± 14.4 blinks/min) was observed at the 275 W stage, achieved by 11 participants. The highest power achieved in the study was 350 W. Only one person reached this stage, and the recorded number of blinks was 22. The longest average eye closure time for individual stages (5.6 ± 4.1 close/s) was observed at the 250 W stage, the final stage reached by all participants. The shortest average eye closure time (4.0 ± 2.6 close/s) was observed during warm-up. Only at the 350 W stage did the average eye closure time fall below 3 s.

Table 2 also shows the average eye closure time divided by the number of blinks for each stage. The average time for a single blink during the whole test oscillated between 0.17 and 0.21 s.

**Figure 5:**
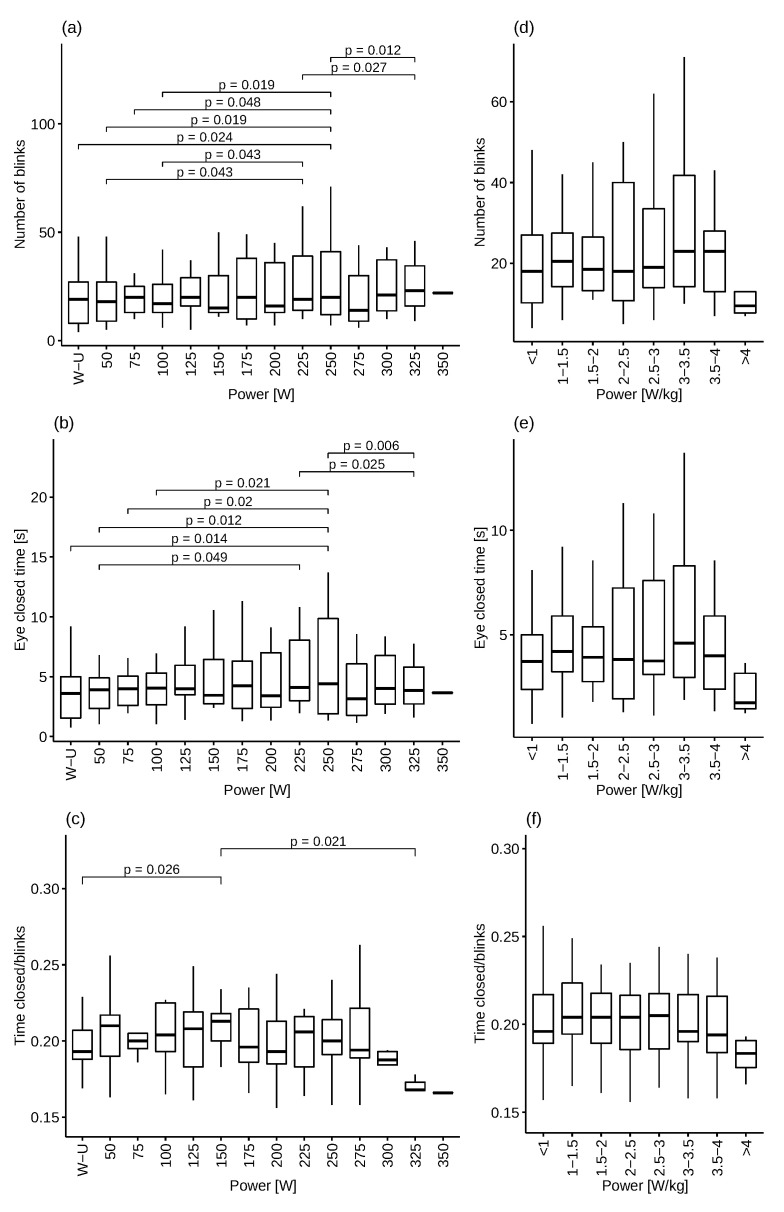
Number of blinks, eye closed time and time closed/blinks.

Figure 5a presents the number of blinks during individual stages and the statistically significant differences between individual stages marked using the Friedman test. The highest average number of blinks was observed during the 250 W stage (27.3 blinks), which was the last stage achieved by all the subjects. Four blinks was the lowest result recorded during the test, observed during the first stage (Warm-up). The largest number of statistically significant differences was observed at the 250 W stage. Statistically significant differences can be observed between the warm-up to 100 W and the 325 W stages. Figure 5d–f show the number of blinks and eye closure times for each stage and single closure times compared to the power/kg of body weight. The highest arithmetic mean of blink numbers was observed with a power-to-weight ratio of 3–3.5 W/kg, while the lowest mean was observed when the power-to-weight ratio was above 4 W/kg.

Figure 5b shows the eye closure time during a given stage and the established statistically significant differences. The highest number of statistically significant differences was also observed for the 250 W stage.

Diagram (c) (Figure 5) shows eye closure time divided by the number of blinks at individual stages. The shortest average time of a single eye closure was observed at the 325 W and 350 W stage, at 0.17 s. The diagram also shows statistically significant differences between individual stages using the Friedman test. Statistically significant differences were observed between the warm-up stage and the 150 W stage, as well as between the 150 W and 325 W stages.

Regarding the blink rate relative to the exercise intensity level, three categories were distinguished, based on the effort intensity relative to the processes of energy metabolism (Table 3). The exercise intensity below 75% HR${}_{max}$ was characterised by the dominance of aerobic processes, at 75 to 90% HR${}_{max}$ mixed processes dominated, and above 90% HR${}_{max}$ anaerobic processes prevailed. The highest blink rate, based on the arithmetic mean, was observed during the exercise intensity of 75 to 90% HR${}_{max}$. For the exercise intensity above 90% HR${}_{max}$, the highest standard deviation was observed, signifying the largest typical area of blink rate variability. The same typical variability area was found during the exercise intensity below 75% HR${}_{max}$, and between 75–90% HR${}_{max}$, when the standard deviation value was 14.4.

Table 3 presents the duration of eye closure relative to three categories of exercise intensity. Based on the arithmetic mean, the longest period of eye closure was 5 s and was observed with a heart rate of 75 to 90% HR${}_{max}$. A similar arithmetic mean value of 4.5 occurred during the exercise intensity below 75% HR${}_{max}$ and above 90% HR${}_{max}$. The maximum period of eye closure (13.7 s) was observed when the exercise intensity was above 90% HR${}_{max}$. Typical variability areas were similar whenever the standard deviation value increased by 0.2 with an exercise intensity of above 90% HR${}_{max}$. In the remaining two categories, the standard deviation value was the same, at 2.8.

The average time of a single blink oscillated between 0.20 and 0.21 s in separate intensity zones. The highest standard deviation (0.2 s) was observed during a stage with an intensity above 90% HR${}_{max}$.

Table 4 shows the verification of the statistically significant differences between HR${}_{max}$ scopes. No statistically significant differences between HR${}_{max}$ scopes were found in any of the analysed parameters.

The next stage of the analysis involved an accurate assessment of the effect of the load, intensity, anthropometric parameters, and body composition on blink frequency and time. The models with the determination ratio $R^{2}$ are presented for the number of blinks (model (1)), eye closure time (model (2)), and single blink time (model (3)) during exercise. The analysis showed that the most powerful effect of variables was observed in the number of blinks with a determination ratio of $R^{2}$ = 0.75. For eye closure time, the determination ratio was $R^{2}$ = 0.69. The simplest structure of the model was observed for a single blink time, where only power, FAT, and BM were included, and the determination ratio was $R^{2}$ = 0.13. Similar structures were calculated for blink frequency and eye closure time, where the models included power, HR, weight, height, FAT, FFM, TBW, BSA, and BMI. It is worth mentioning that statistical significance has been observed for each determination ratio. An analysis using multiple regression forward selection allowed for the determination of the optimum system of variables for individual models.

(1) $$\begin{array}{cc} \mathrm{Blinks}\;\mathrm{Eye} & =-368-0.1\cdot P+0.4\cdot \mathrm{HR}-5.7\cdot W\;+12.2\cdot H+24.3\cdot \mathrm{FAT}\\ \; & +28.7\cdot \mathrm{FFM}+10.5\cdot \mathrm{TBW}-1981.5\cdot \mathrm{BSA}-9.6\cdot \mathrm{BMI} \end{array}$$

(2) $$\begin{array}{c} \mathrm{Time}\;\mathrm{of}\;\mathrm{closed}\;\mathrm{eye}=-79.9-0.02\cdot P+0.07\cdot \mathrm{HR}-1.4\cdot W+2.3\cdot H\\ +4.6\cdot \mathrm{FAT}+5.6\cdot \mathrm{FFM}+2\cdot \mathrm{TBW}-362.4\cdot \mathrm{BSA}-1.6\cdot \mathrm{BMI} \end{array}$$

(3) $$\begin{array}{cc} \mathrm{Time}\;\mathrm{of}\;\mathrm{one}\;\mathrm{blink} & =0.2-0.01\cdot P-0.01\cdot \mathrm{FAT}+0.01\cdot \mathrm{BMI} \end{array}$$

## 5. Discussion

The changes in adult physiology that occur during the transition from resting to stationary aerobic exercise are well known [32]. Improved physiological eye function is demonstrated through sporting activities, and sport is considered essential in the prevention of common eye diseases [10]. The relationship between eye blink frequency and incremental exercise is relatively unknown. Recent findings include that spontaneous eye blinking is the missing link between aerobic fitness and cognitive function [23]. In our study, blink frequency and eye closure time were determined by a group of somatic and physiological factors. These variables are worth considering in the future assessment of fatigue induced by exercise and its relationship with an athlete’s cognitive processes in order to prevent a decline in psychomotor performance during incremental exercise [33].

The measurements in the present study were intended to verify a potential relationship between blink rate and increasing fatigue during progressive aerobic exercise on a cycle ergometer. The results were analysed and suggest that increasing workload did not affect the blink rate. The mean blink rate during all stages of the progressive test was 20 to 38 blinks per minute. Eye closure time at individual stages oscillated between 3.7 and 5.6 s. In a study by Bentivoglio et al. [3], blink rates were analysed during various activities; 150 subjects, aged 5 to 87 years, were recorded while performing three activities: reading, talking, and resting. The highest blink rate was observed during a conversation, at a mean value of 26. The mean blink rate while resting was 17. The lowest blink rate, at a mean of 4.5 blinks, was observed while reading. The results suggest that cognitive processes decrease the blink rate. Magliacano et al. [34] showed that when visual attention is not required during an auditory cognitive task then spontaneous eye blinking increases. Another study involved 25 subjects, with the measurement of the blink rate per minute in subjects playing a fast-paced game, a slow-paced game, and while resting. The mean blink rate while resting was 24.36. In subjects playing the slow-paced game, the number of blinks was reduced nearly by half, with a mean blink rate of 12.44, whereas during the fast-paced game it was 8.96. These results indicate that during the performance of cognitive tasks, the blink rate decreases along with increases in the visual presentation rate [35]. The relationship between blink rate and cognitive tasks was examined in a test comparing the blink rate while resting and while reading or memorising. The results indicate that during a reading session, the blink rate is lower and suggests that the blink rate is affected by the type of intellectual activity [36].

The outcomes were compared considering four categories of load (expressed in watts) per kilogram of body weight. The results did not indicate a statistically significant correlation. For each category of load per kilogram of body weight, the average number of blinks was 20. The lowest average blink number was observed for power above 4 W/kg. There was no relationship between eye closure time and W/kg rate. Similarly, the study carried out by Kim et al. [37] did not show a relationship between blink rate and tiredness. The study involved an analysis of the blink rate based on the position of the pupil while watching a stereoscopic film, using an infra-red camera and an infra-red light source. The final results show high variability in the blink rate over time; therefore, they are not consistent with those studies that indicate an increased blink rate associated with fatigue [20,23,28]. Another study [38] involved a measurement of the number of blinks during a conversation via a camera mounted on the face. The mean blink rate in subjects during a conversation was 16.4 blinks. During a test conducted at noon, the mean number of blinks was 4.98, whereas for a test at midnight, expected to reveal drowsiness, the mean blink rate was 3.33 per minute. It is believed that if the blink rate decreases below 3 per minute, it may indicate a state of insomnia [38]. Haq and Hasan [16] analysed the effect of the level of attentiveness and fatigue on the number of blinks. They assumed that fatigue and reduced attentiveness affected the blink rate, compared to the baseline values. When the number of blinks varied from the threshold value, due to fatigue of lack of attentiveness, the driver received a warning signal about the danger. The threshold value adopted in the test was 8–10 blinks per minute. The reduced number of blinks may result from longer periods of eye closure, possibly due to dozing off [16]. However, Sigari et al. [39] claim that a significant reduction in the blink rate may result from inattentiveness, whereas an increased blink rate compared to the norm may be indicative of driver fatigue. The method of identifying fatigue with the use of eye-blinking analysis was also used in a study by Benedetto et al. [40]. The results indicate that blink duration is a more sensitive and reliable indicator of driver workload than blink rate. The study did not provide significant results regarding the number of blinks, due to the high individual variability, which may indicate that the blink rate is a complex variable, affected by numerous factors.

The level of fatigue was also presented compared to the heart rate. The division by aerobic values distinguished three categories of aerobic exercise: aerobic, mixed, and anaerobic, based on the ratio of the percentage heart rate compared to HR${}_{max}$. The results for the blink rate, eye closure time, and a single blink were similar on every level of HR${}_{max}$. Forward selection multiple regression models showed that blink rate and eye closure time depend on the load, intensity, HR, weight, height, FAT, FFM, TBW, BSA, and BMI. The analysis confirms that blink frequency and eye closure time depend on many variables, not on one factor. To examine the relationship between the number of blinks and increased physical effort, Khanal et al. [28] conducted tests on three subjects exercising on a cycle ergometer with increasing workload. The pre-test stage was a 5-min warm-up, followed by the first stage at 75 W, and then the power was increased by 15 W per minute. The camera recording blinks were placed on a stand in front of the tested subject. The results demonstrate that the blink rate increases along with the perceived fatigue of the organism. The analysis presented in our study does not support this thesis as no correlation was determined between the blink rate and fatigue. It should be noted that in our study, the test group was larger. The test group of Khanal et al. [28] consisted of 3 people. Marandi et al. [41] showed that with fatigue, blink frequency and eye closure time increases, regardless of age. Other studies revealed that during a more intense concentration on a cognitive task, blink frequency is lower [4,6].

Unfortunately, the results of different studies are not unambiguous as the blink rate may be affected by numerous environmental factors. The limited number of studies on the correlation between blink rate and fatigue also prevents the formulation of definite conclusions.

The limitations of the study were related to the small number of participants in the study group. Moreover, the study was conducted only on a group of Rzeszow University students who were not professional athletes. In the future, the study may be extended to include measurements of a wider group of sports and non-sports people. The test was performed only on a cycle ergometer, but the study can be extended to include measurements on a treadmill, focusing on athletes whose discipline is characterised by running and not cycling. The study can be extended to analyse and measure metabolic parameters such as VO${}_{2}$, VCO${}_{2}$, minute ventilation, and energy expenditure to find the relationship between these parameters, blink frequency, and eye closure time.

## 6. Conclusions

The analysis of the data led to the following conclusions:

(1) The highest number of statistically significant differences in the blink rate and eye closure time was observed at the 250 W stage. At the same time, no statistically significant differences in relation to the load calculated in Watts per kilogram of body weight were observed.
(2) The analysis showed no significant differences in blink rate, eye closure time, and single blink time in terms of heart rate ranges, during the experiment on a cycle ergometer.
(3) The analysis of regression models revealed that the blink rate and eye closure time were determined by a group of factors. For the blink rate and eye closure time, the following factors were important: the value of cycle ergometer load power, heart rate, body weight, adipose tissue mass, fat-free mass, and total body water and body surface ratio, while for a single blink time factors such as cycle ergometer load power, adipose tissue mass and body weight ratio were important.

## Figures and Tables

**Table 1:** Characteristic of participants.

	$\bar{x}$	sd	min	max
Body weight [kg]	75.9	10.1	54.8	91.8
Body height [cm]	176.8	6.4	167	186
BMI [kg/m${}^{2}$]	24	3.6	17.1	29.7
FAT [%]	13	5.8	3.2	20.6
FFM [kg]	65.6	6.3	53	76.1
TBW [kg]	44.6	3.7	37.1	50.2
HR${}_{max}$ [bpm]	182.2	10	161	193
HR${}_{rest}$ [bpm]	87.8	9.9	76	106
Power${}_{\bar{x}}$ [W/kg]	2.3	1.2	0.5	5.5
Power${}_{max}$ [W/kg]	3.9	0.6	3.1	5.5
BSA [m${}^{2}$]	1.92	0.13	1.62	2.1

BMI—Body Mass Index, FAT—body fat mass, FFM—fat-free body mass, TBW—total body water, HR—Heart rate, Power${}_{\bar{x}}$—average power, BSA—Body Surface Area, ${}_{\bar{x}}$—mean value, sd—standard deviation, min—minimum value, max—maximum value.

**Table 2:** Characteristic of analysed parameters in term of power.

Power [W]	N	Blinks Eye [n]		Time of Closed Eye [s]		Time/Closed [s/n]	
		$\bar{x}\pm \mathrm{sd}$	Me	$\bar{x}\pm \mathrm{sd}$	Me	$\bar{x}\pm \mathrm{sd}$	Me
Warm up	13	20.5 ± 14.6	19.0	4.0 ± 2.6	3.6	0.2 ± 0.03	0.19
50	13	20.5 ± 12.8	18.0	4.1 ± 2.5	3.9	0.21 ± 0.03	0.21
75	13	22.5 ± 13.4	20.0	4.5 ± 2.4	4.0	0.2 ± 0.03	0.2
100	13	21.3 ± 13.1	17.0	4.4 ± 2.5	4.1	0.21 ± 0.03	0.2
125	13	25.2 ± 16.2	20.0	5.1 ± 2.9	4.0	0.21 ± 0.03	0.21
150	13	23.2 ± 13.5	15.0	4.9 ± 2.9	3.5	0.21 ± 0.02	0.21
175	13	23.2 ± 14.7	20.0	4.7 ± 3.1	4.3	0.2 ± 0.02	0.2
200	13	22.5 ± 13.8	16.0	4.5 ± 2.8	3.4	0.2 ± 0.03	0.19
225	13	26.4 ± 17.4	19.0	5.3 ± 3.2	4.1	0.21 ± 0.03	0.21
250	13	27.3 ± 20.0	20.0	5.6 ± 4.1	4.4	0.2 ± 0.02	0.2
275	11	19.8 ± 14.4	14.0	4.1 ± 2.9	3.2	0.21 ± 0.04	0.19
300	6	24.8 ± 14.4	21.0	4.7 ± 2.7	4.0	0.19 ± 0.02	0.19
325	3	26.0 ± 18.7	23.0	4.4 ± 3.1	3.9	0.17 ± 0.01	0.17
350	1	22.0	22.0	3.7	3.7	0.17	0.17

${}_{\bar{x}}$—mean value, sd—standard deviation, Me—Median.

**Table 3:** Characteristic of analysed parameters in term of heart rate ranges.

% HR${}_{\max}$	Blinks Eye		Time of Closed Eye		Time/Closed	
	$\bar{x}\pm \mathrm{sd}$	Me	$\bar{x}\pm \mathrm{sd}$	Me	$\bar{x}\pm \mathrm{sd}$	Me
R1—≤75%	22.3 ± 14.4	18.0	4.5 ± 2.8	3.9	0.21 ± 0.03	0.20
R2—75–90%	25.1 ± 14.4	21.0	5.0 ± 2.8	4.0	0.20 ± 0.03	0.20
R3—≥90%	22.6 ± 15.3	19.0	4.5 ± 3.0	3.8	0.20 ± 0.2	0.19

R1—≤75% HR${}_{max}$, R2—75—90% HR${}_{max}$, and R3—≥90% HR${}_{max}$.

**Table 4:** Differences between analysis parameters in terms of heart rate ranges.

Variable	R1 vs. R2	R1 vs. R3	R2 vs. R3
	*p*	*p*	*p*
Blinks Eye	0.9166	0.8489	0.9497
Time of closed eye	0.9280	0.9961	0.9713
Time/closed	0.6721	03612	0.2877

R1—≤75% HR${}_{max}$, R2—75—90% HR${}_{max}$, and R3—≥90% HR${}_{max}$.

## Data Availability

Samples of the compounds are available from the authors.
